# Transient Shifts in Bacterial Communities Associated with the Temperate Gorgonian *Paramuricea clavata* in the Northwestern Mediterranean Sea

**DOI:** 10.1371/journal.pone.0057385

**Published:** 2013-02-20

**Authors:** Marie La Rivière, Marie Roumagnac, Joaquim Garrabou, Marc Bally

**Affiliations:** 1 Mediterranean Institute of Oceanography (MIO) UM 110, CNRS/INSU, IRD, Aix-Marseille Université, Université du Sud Toulon-Var, Marseille, France; 2 Institut de Ciències del Mar (ICM), CSIC, Barcelona, Spain; Universidade Federal do Rio de Janeiro, Brazil

## Abstract

**Background:**

Bacterial communities that are associated with tropical reef-forming corals are being increasingly recognized for their role in host physiology and health. However, little is known about the microbial diversity of the communities associated with temperate gorgonian corals, even though these communities are key structural components of the ecosystem. In the Northwestern Mediterranean Sea, gorgonians undergo recurrent mass mortalities, but the potential relationship between these events and the structure of the associated bacterial communities remains unexplored. Because microbial assemblages may contribute to the overall health and disease resistance of their host, a detailed baseline of the associated bacterial diversity is required to better understand the functioning of the gorgonian holobiont.

**Methodology/Principal Findings:**

The bacterial diversity associated with the gorgonian *Paramuricea clavata* was determined using denaturing gradient gel electrophoresis, terminal-restriction fragment length polymorphism and the construction of clone libraries of the bacterial 16S ribosomal DNA. Three study sites were monitored for 4 years to assess the variability of communities associated with healthy colonies. Bacterial assemblages were highly dominated by one *Hahellaceae*-related ribotype and exhibited low diversity. While this pattern was mostly conserved through space and time, in summer 2007, a deep shift in microbiota structure toward increased bacterial diversity and the transient disappearance of *Hahellaceae* was observed.

**Conclusion/Significance:**

This is the first spatiotemporal study to investigate the bacterial diversity associated with a temperate shallow gorgonian. Our data revealed an established relationship between *P. clavata* and a specific bacterial group within the *Oceanospirillales*. These results suggest a potential symbiotic role of *Hahellaceae* in the host-microbe association, as recently suggested for tropical corals. However, a transient imbalance in bacterial associations can be tolerated by the holobiont without apparent symptoms of disease. The subsequent restoration of the *Hahellaceae*-dominated community is indicative of the specificity and resilience of the bacteria associated with the gorgonian host.

## Introduction

The interactions between microbial communities and sessile marine invertebrates such as sponges or corals are increasingly recognized as a critical component of the overall biology of these organisms. Recent research on the microbiology of scleractinian corals has demonstrated close relationships between the hexacoral animal and numerous prokaryotic organisms, and studies on coral reef ecosystems should consider the coral host with its associated microbiota as a unique evolutionary unit termed the “holobiont” [1]–[3]. Corals harbor highly diverse bacterial communities, and several reports have highlighted the existence of specific coral-bacteria associations that are mostly maintained among colonies from the same locality or even across distinct geographical locations [2], [4]–[7]. These bacterial assemblages may play important roles in the host's physiology, mainly through their functions in nutrient cycling and health status [8]. In particular, there is increasing evidence for a relationship between coral-associated bacteria and disease resistance, which is likely the result of antibiotics produced by the resident microbiota and/or niche competition with potential pathogens [1], [9], [10].

In contrast to scleractinian reef-forming corals, information on the microbial diversity associated with gorgonians (octocorals) is limited. A few studies have investigated bacterial associations with tropical or cold-water species [11]–[13], but to our knowledge, the bacterial diversity of shallow temperate octocorals has not yet been explored. In the Northwestern (NW) Mediterranean Sea, gorgonians play an important ecological role in highly diverse coralligenous outcrops [14]. During the last few decades, coralligenous benthic invertebrates have suffered from large-scale disease and mortality [15]–[18]. These events are linked to unusual positive anomalies of seawater temperature in the summer and have affected a wide range of macro-benthic species over hundreds of kilometers, from the Italian to Spanish coasts [19]. Gorgonians populations have undergone extensive damage, although differences in the degree of impact have been observed in various geographic areas.

One of the most affected species is the red gorgonian *Paramuricea clavata* (Risso, 1826), a long-lived, aposymbiotic colonial octocoral that is considered a key species within coralligenous assemblages [20]. During the summer mortality outbreaks, *P. clavata* colonies exhibited symptoms of necrosis that may have been caused by multiple factors acting in synergy with thermal stress, including food limitation, metabolic constraints and microbial virulence [15], [21]. Colonies that were affected during mass mortality events in 2003 and 2008 harbored culturable isolates of *Vibrio coralliilyticus*, a pathogenic bacterium that can induce host tissue damage at elevated temperatures [22], [23]. Members of this *Vibrio* clade were previously identified as the etiologic agents of bleaching in the Indo-Pacific coral *Pocillopora damicornis* and White Syndrome disease in other coral species, thus satisfying Koch's postulate [24], [25]. This finding suggests that at least some mechanisms underlying the disease process in *P. clavata* are comparable to the mechanisms involved in tropical coral outbreaks. Several studies have concluded that coral pathogens take advantage of changes in the bacterial community structure during stressful conditions to proliferate and cause tissue damage [4], [26], [27]. In support of this hypothesis, compositional shifts in bacterial assemblages have been observed in diseased coral colonies [28], [29]. Similarly, the recurrent disease and mortality of *P. clavata* may be promoted by a disturbance of the resident microbiota in response to anomalous high-temperature conditions during the summer.

To evaluate the contribution of microbial communities to temperate gorgonian health and disease, a detailed knowledge of the structure of the bacterial assemblages in healthy colonies is required. The objective of the present study was to provide a baseline of the bacterial communities associated with *P. clavata* populations in the NW Mediterranean basin. Given the predicted increase in extreme climatic events in this region, new mortality outbreaks are expected [30], [31]. Thus, tracking potential modifications of this baseline during future high-temperature stress could greatly aid our understanding of the role of bacterial associations in maintaining gorgonian health.

In this work, we conducted a seasonal sampling over 4 years (2007–2010) in 3 distinct geographical locations separated by hundreds of kilometers (Provence, Corsican and Catalan coasts), which allowed us to characterize the bacterial communities associated with *P. clavata* and the spatiotemporal variability of their structure. Three different molecular culture-independent approaches (denaturing gradient gel electrophoresis (DGGE), terminal-restriction fragment length polymorphism (T-RFLP) and 16S RNA gene clones library construction) were used to determine which bacteria might be conserved across geographically remote *P. clavata* populations. Our findings reveal the presence of host-specific bacterial associations in the gorgonian holobiont, although transient and reversible variations in microbiota composition were also recorded during our seasonal survey.

## Materials and Methods

### Ethics Statement

The sampling in the Mediterranean Sea was performed in accordance with French professional diving rules and did not involve endangered or protected species. Annual sampling permits for the site located in Riou (France) were delivered by the *Préfet de la region Provence-Alpes-Côte d'Azur*. Specific permits delivered by the *DIRM Méditerranée,* advised by the *Conseil Scientifique de la Réserve Naturelle de Scandola* and the *Departament d'Agricultura, Ramaderia, Pesca, Alimentació i Medi Natural de la Generalitat de Catalunya,* were obtained to sample gorgonian tissues in the marine protected areas of Scandola (Corsica) and Medes Islands (Spain). Gorgonian sampling entailed the collection of only a few centimeters of the apical part of the branches. Because gorgonians are colonial organisms, the sampling caused unnoticeable injuries that healed rapidly after the collection.

### Sample collection and processing

**Figure 1:**
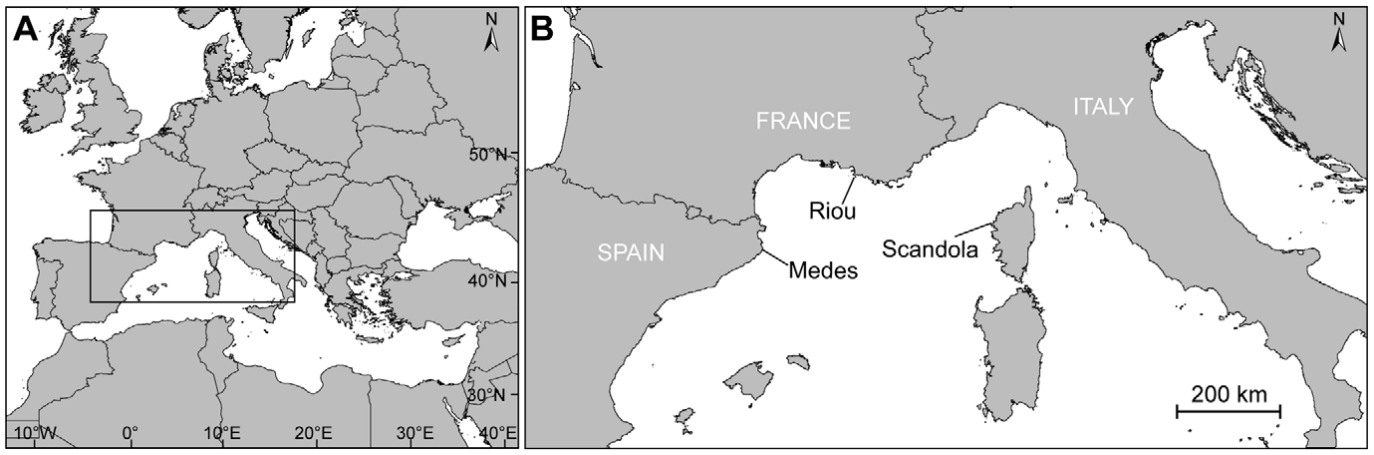
Sampling sites. Map of the Northwestern Mediterranean basin (A) and an enlargement showing the location of the study sites (B).

Samples of *P. clavata* were collected from 3 sites in the NW Mediterranean Sea, Riou Island (France) (43°10.345′ N, 05°23.319′ E), Scandola (France) (42°22.793′ N, 8°33.000′ E) and Medes Island (Spain) (42°2.882′ N, 3°13.579′ E) (Figure 1), at an approximately 20 m depth in winter (January to March) and summer (June to September) from 2007 to 2010. Apical branch fragments (2 cm length) of randomly chosen, apparently healthy colonies (i.e., with no visible signs of necrosis) (n = 3) were collected using shears and placed in plastic bags underwater. Three liters of surrounding seawater were also collected in plastic bottles. The collected samples were then transferred to the Mediterranean Institute of Oceanography within 2 h (Riou site) or frozen on dry ice for subsequent transportation to the laboratory (Scandola and Medes sites).

The *P. clavata* samples were rinsed 3 times with sterile 0.22 µm-filtered seawater to remove loosely associated bacteria. Tissues were detached from the central axis and gently crushed using sterile forceps and a scalpel blade in 3 ml of sterile seawater. The tissue slurry was homogenized and aliquoted into 3 microtubes. Samples were then spun at 13,000 g, and the supernatant was removed before storing the tissue pellets at −80 °C. The surrounding seawater samples were immediately filtered through 0.22 µm Sterivex® filter units (Merck Millipore, Billerica, MA, USA). The filters were stored at −80 °C for subsequent DNA extraction.

### DNA extraction

Bacterial DNA in gorgonian tissue samples was extracted following the protocol of Bourne *et al.* [28]. Briefly, tissue slurries were resuspended in 0.5 ml extraction buffer (50 mM Tris-HCl pH 8.0, 40 mM EDTA, 0.75 M sucrose) with 1 µg of salmon sperm DNA and incubated for 5 min at room temperature. Next, a lysozyme solution (75 µl of 100 mg·ml^−1^) was added. The samples were incubated for 1 h at 37 °C with slow agitation and submitted to 3 freeze-thaw cycles (ranging from −80 °C to 70 °C); these steps were followed by the addition of 100 µl 25% sodium dodecyl sulfate (SDS) and a further incubation of 10 min at 70 °C. The samples were digested by proteinase K (20 µl of 20 mg·ml^−1^) for 1 h at 37 °C with slow agitation. The tissue lysates were again submitted to 3 freeze-thaw cycles and extracted by standard phenol-chloroform DNA extraction procedures. After precipitation with 50 µl 3 M sodium acetate and an equal volume of isopropanol, the DNA was pelleted by centrifugation (16,000 g for 30 min at 4 °C), washed with cold 70% ethanol and suspended in 30 µl sterile ultra-pure water. The DNA concentration was estimated by spectrophotometry using a biophotometer (Eppendorf, Hamburg, Germany), and the DNA samples were stored at −20 °C until further processing.

To isolate DNA from seawater samples, Sterivex® filter units were filled with 500 µl of lysis buffer (50 mM Tris-HCl pH 8, 100 mM NaCl, 1% SDS) and 12.5 µl proteinase K (20 mg·ml^−1^). After an overnight incubation at 55 °C, the cell lysate was recovered from the filter unit, and total DNA was extracted by standard phenol-chloroform extraction procedures. The DNA in the liquid phase was then precipitated with ethanol and concentrated by centrifugation at 16,000 g for 10 min at 4 °C. The DNA pellets were washed with 70% cold ethanol, resuspended in 100 µl sterile ultra-pure water and stored at −20 °C until analysis.

### DGGE analysis

#### PCR amplification

Bacterial 16S rDNA were amplified from genomic DNA extracted from *P. clavata* colonies and seawater using 4 different pairs of universal bacterial primers targetting various hypervariable regions (V) of 16S rRNA gene: 27F/536R (V1–V3) [32], EUB f933/EUB r1387 (V6–V7) [33], 341F/907R (V3–V5) [34] and 341F/907RA (V3–V5) [35]. A 40-bp GC-rich sequence (GC-clamp) was attached to the 5′ end of the forward primers to enhance separation in DGGE [34].

PCR (35 cycles) was performed on a Mastercycler® thermocycler (Eppendorf) in a total volume of 50 µl containing 1 X Taq® Flexi Buffer (Promega, Fitchburg, WI, USA), along with 1.5 mM MgCl_2_, 200 µM of each deoxynucleoside triphosphate (dNTP), 1 µM forward and reverse primer, 0.3–3 µl (approximately 100–300 ng) of bacterial DNA template, 1 U of GoTaq® Flexi DNA Polymerase (Promega), and sterile ultra-pure water. After an initial denaturation of 1 min at 96 °C, a touchdown PCR protocol was performed in which the primer annealing temperature was initially set at 53 °C (27F/536R), 62 °C (EUB f933/EUB r1387) or 58 °C (341F/907R and 341F/907RA) and was decreased by 0.5 °C with each cycle of the next 20 cycles. The final annealing temperature was then maintained for the remaining 15 cycles of the reaction. For each cycle, denaturation was performed at 94 °C for 1 min, followed by annealing for 1 min and extension at 72 °C for 1 min. The PCR products were evaluated by electrophoresis on 1% agarose gels stained with ethidium bromide.

#### Electrophoresis conditions and DGGE band analysis

DGGE of the PCR products was performed using the DCode® system (Bio-Rad, Hercules, CA, USA) on a 6% (wt/vol) polyacrylamide gel with a linear denaturing gradient of 35% to 60% in 1 X Tris-acetate-EDTA buffer (TAE). Electrophoresis was performed for 17 h at 60 °C at a constant voltage of 80 V. The gels were stained with GelStar® (Cambrex BioScience, East Rutherford, NJ, USA) in 1 X TAE and visualized under UV light with a Gel Doc 2000® image system (Bio-Rad).

To identify the 16S rDNA bacterial sequences, selected DGGE bands were excised from the gels and eluted overnight at 4 °C in 50 µl sterile ultra-pure water. PCR re-amplification was performed with the corresponding primer set. The PCR products were purified with Wizard® PCR Clean-Up minicolumns (Promega), and the recovered 16S rDNA gene fragments were sequenced (Eurofins MWG Operon, Ebersberg, Germany).

### T-RFLP analysis

#### PCR amplification, enzymatic digestion and T-RFLP

For T-RFLP analysis, the bacterial 16S rRNA genes were amplified by PCR with the extracted DNA using the universal primers 63F [36] and 786R-Eub (a reverse-complement version of 768F-Eub; [37]). This primer pair target the V1–V4 hypervariable regions of the 16S rRNA gene. The forward primer was labeled at the 5′ end with phosphoramidite fluorochrome 6-carboxyfluorescein (Applied Biosystems, Carlsbad, CA, USA). PCR amplifications were performed in a total volume of 25 µl containing 1 X Taq Buffer with (NH_4_)_2_SO_4_ (Fermentas, Burlington, Canada), 200 µM each dNTP, 1 µM forward and reverse primer, 2 mM MgCl_2_, 0.1 µg µl^−1^ bovine serum albumin (BSA), 0.3–3 µl (approximately 100–300 ng) bacterial DNA template, 1 U native Taq DNA Polymerase (Fermentas) and sterile ultra-pure water. After an initial denaturation step (5 min at 94 °C), 35 amplification cycles (94 °C for 1 min, 48 °C for 1 min, 72 °C for 1 min) were performed on a Mastercycler® thermocycler (Eppendorf). Amplification was confirmed by migration on a 1% agarose gel in TAE and ethidium bromide staining. The labeled PCR products were purified using the Wizard® PCR Clean-Up System (Promega) following the manufacturer's instructions and eluted in 30 µl of DNase-free distilled water (Promega).

The restriction reactions were performed for 16 h at 37 °C using a digestion mixture containing 6 µl of the purified PCR products, 1 X reaction buffer (Promega), 0.1 µg µl^−1^ BSA and 20 U *Cfo*I or *Msp*I (Promega) in a final volume of 20 µl. Each digested sample (3 µl) was mixed with 0.3 µl GeneScan 600-LIZ® (Applied Biosystems) size standard and 10 µl Hi-Di® Formamide (Applied Biosystems) in a 96-well plate and denatured at 90 °C for 3 min. The labeled terminal restriction fragments (TRFs) were then separated on an ABI 3130 Genetic Analyzer (Applied Biosystems) with default fragment analysis parameters. Each sample was run twice as replicates to account for migration discrepancies. All T-RFLP electropherograms were visually inspected for possible artifacts and incorrect peak determination and were tabulated in GeneMapper® version 4.0 software (Applied Biosystems) using the Local Southern method as the size-calling algorithm [38]. Only fragments longer than 80 bp and smaller than 600 bp were considered.

#### Statistical analysis of the spatiotemporal variability

The raw data sets (peak sizes in base pairs and peak areas in fluorescence units) for the 2 restriction enzymes were exported to Excel (Microsoft, Redmond, WA, USA). To produce a single composite profile for each sample, the average area and size for each peak were calculated between duplicates (TRFs with sizes within 0.5 bp of each other were considered identical, while TRFs that were present in only one of the duplicates were removed). Because the amount of DNA template loaded for T-RFLP analysis could not be controlled accurately, T-RFLP profiles were normalized by applying the variable percentage threshold, as described by Osborne *et al.* [39]. To eliminate background fluorescence, only peaks contributing to more than 1.2% of total fluorescence (according to the variable percentage threshold result) were considered. Data were binned using the interactive and automatic binning algorithms [40] implemented in the free R programming language [41], applying custom R binning scripts (WS 1; Sh 0.1) to account for size calling imprecision. Based on the “best binned frame” identified by the algorithm, the data were transformed for further graphical and statistical representations. The relative abundances in the different analyzed samples were calculated for each TRF. The data were transformed to a binary matrix (presence/absence), and the distance matrix between samples was computed from the Dice similarity index using R (R package *ade4*; [42]). Patterns of bacterial community similarities were represented on a 2-D scatter plot using multidimensional scaling (MDS) ordination. The K-means clustering method was then performed to determine the center coordinates of the clusters (R package *fpc*; [43]). Confidence ellipses were superimposed on the MDS ordination plot for each previously determined cluster, with 2 confidence levels of 50% and 95% (R package *car*; [44]).

### Bacterial clone library construction

The universal bacterial primers 63F and 1389R [36] were used to amplify nearly full-length 16S rRNA genes from the gorgonian tissue samples. The PCR mixtures contained 1 X of Taq® Flexi Buffer (Promega), 1.5 mM MgCl_2,_ 200 µM each dNTP, 1 µM each primer, 1 U GoTaq® Flexi DNA polymerase (Promega) and 0.3–3 µl (approximately 100–300 ng) DNA template in a total volume of 25 µl. PCR was performed with an initial 5 min denaturation step at 94 °C, followed by 35 amplification cycles consisting of 94 °C for 1 min, 55 °C for 1 min and 72 °C for 1 min. The final elongation step was performed at 72 °C for 10 min. For each library, the DNA extracted from 3 *P. clavata* colonies was PCR-amplified in 3 separate reactions, each performed in triplicate. The amplified products were pooled and purified with Wizard® PCR Clean-Up minicolumns (Promega), followed by cloning into the pGEM®-T Easy vector (Promega) according to the manufacturer's instructions. After transformation into *E. coli* JM109 competent cells, the orientation of the inserted 16S rRNA gene of each resulting clone was determined by PCR re-amplification using 63F and M13 reverse primers. Clones producing a PCR product of the expected size (approximately 1,500 bp) were sequenced with a plasmid forward primer (LGC Genomics GmbH, Berlin, Germany). A total of 700 partial 16S rRNA gene sequences were obtained. For the Riou site, 3 libraries were constructed from samples collected in winter 2007 (93 clones), summer 2007 (176 clones), and winter 2008 (84 clones). One library per site was constructed from samples collected in summer 2007 for Medes (171 clones) and Scandola (176 clones).

### Phylogenetic analysis

All bacterial 16S rDNA clone sequences were trimmed to 750 bp starting at the 5′ end of the 63F primer, and the production of chimeras during PCR was evaluated with the Pintail program [45]. The resulting libraries were dereplicated using FastGroupII ([46]; http://biome.sdsu.edu/fastgroup/index.htm) with a similarity cutoff value of 97% for sequence group definition. The unique sequence groups were classified taxonomically at the family level by the SeqMatch tool of the Ribosomal Database Project (RDP; http://rdp.cme.msu.edu). Within the dominant family-level groups, the sequence affiliations and closest relatives were identified by comparison with the GenBank database using the BLAST algorithm ([47]; http://blast.ncbi.nlm.nih.gov/Blast.cgi). To describe the compositional differences between the 3 summer 2007 libraries, the Shannon-Weaver index for diversity [48] and the Chao1 richness index [49] were calculated using the RDP Pipeline tools, with sequence groups defined with 97% similarity.

The consistency between the identified ribotypes in the libraries and the major TRFs in each community's peak profiles was confirmed by T-RFLP analysis of representative 16S rDNA clones of dominant ribotypes. Partial sequence reads obtained from the excised DGGE bands were taxonomically assigned by BLAST analysis against the GenBank database.

### Nucleotide sequences accession numbers

The nucleotide sequence data for the partial 16S rRNA genes reported in this paper were deposited in GenBank under the accession numbers JX874190-JX874360 (Medes; summer 2007 library), JX874361-JX874536 (Riou; summer 2007 library), JX874537-JX874629 (Riou; winter 2007 library), JX874630-JX874713 (Riou; winter 2008 library) and JX874714-JX874889 (Scandola; summer 2007 library).

## Results

### DGGE fingerprinting of *P. clavata*-associated bacterial communities

**Figure 2:**
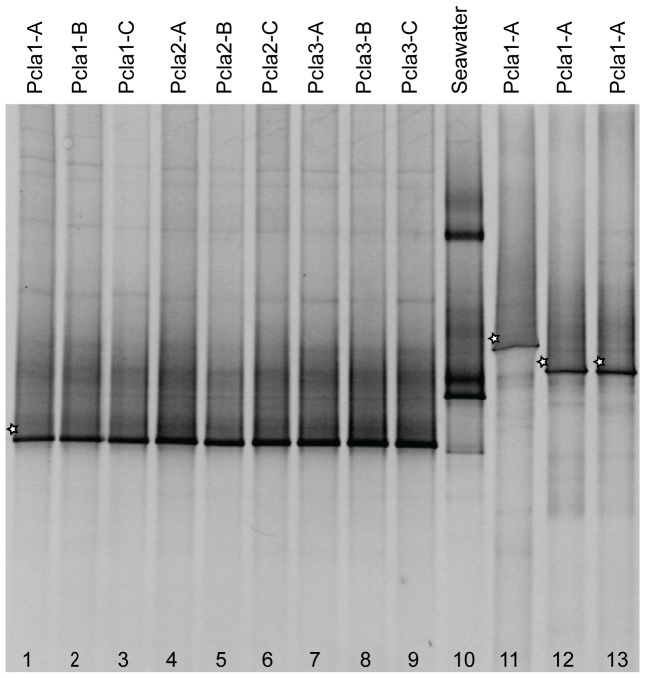
Representative denaturing gradient gel electrophoresis (DGGE) profiles of the bacterial community composition. DGGE analysis of bacterial communities associated with triplicate samples (A, B and C) of 3 *P. clavata* colonies (Pcla1 to Pcla3) and the surrounding seawater sampled in Riou in winter 2007. 16S rDNA was amplified with different sets of primers: 27F-GC/536R (lanes 1 to 10), EUB f933-GC/EUB r1387 (lane 11), 341F-GC/907RA (lane 12) and 341F-GC/907R (lane 13). The sequenced DGGE bands are indicated with a star.

To estimate the intra- and inter-colonial variability of bacterial community composition, apical branches from *P. clavata* colonies sampled in Riou in winter 2007 were analyzed by 16S rDNA-DGGE fingerprinting. PCR amplification with 27F-GC/536R universal primers followed by denaturing electrophoresis yielded repeatable banding patterns for all 3 fragments sampled from each colony (Figure 2, lanes 1–9). The consistency of the banding patterns from different *P. clavata* colonies was clearly apparent, indicating the presence of similar bacterial diversity. By contrast, the DGGE profile of seawater collected in close vicinity of the colonies (Figure 2, lane 10) was markedly different and did not share detectable bands with the *P. clavata* tissue samples. The dominant 16S rDNA band in the fingerprint of *P. clavata* was excised from the gel and sequenced for identification of the corresponding ribotype. This band was related to 16S rDNA sequences from marine bacteria belonging to the order *Oceanospirillales*. The closest cultivated relative was *Spongiobacter nickelotolerans* (GenBank accession number AB205011), with 94% similarity along the 479 bp sequence length.

The dominant DGGE bands obtained with 3 other universal bacterial primer pairs were also sequenced. The band obtained with the EUB f933/EUB r1387 primer pair (Figure 2, lane 11) was related to *Spongiobacter nickelotolerans* 16S rDNA (94% similarity among 417 bp). The sequences recovered from the 341F-GC/907RA and 341F-GC/907R amplifications (Figure 2, lanes 12–13) shared 91 to 92% similarity with the 16S rDNA of *Endozoicomonas elysicola* (AB196667), a close relative of *Spongiobacter* spp. This similarity indicated that the predominance of single bands observed in all the *P. clavata* DGGE profiles was not caused by amplification biases but likely resulted from an abundant population of ribotypes belonging to the *Oceanospirillales* order.

### T-RFLP analysis of bacterial community variation

**Figure 3:**
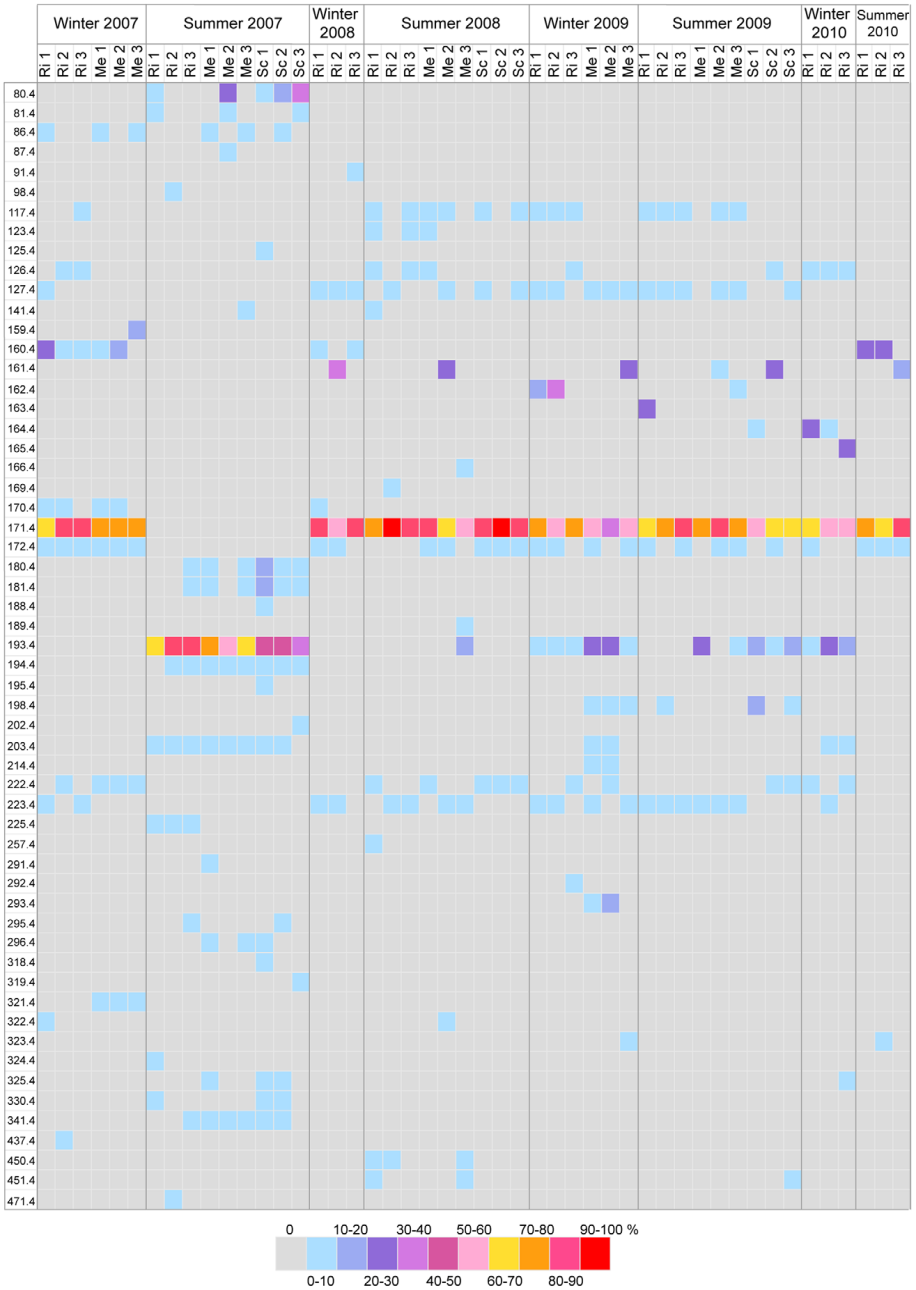
Relative abundances of *Cfo*I TRFs in *P. clavata* samples analyzed by T-RFLP. Each row represents a TRF generated by *Cfo*I digestion of PCR amplicons from samples that were collected from the different sites (Riou (Ri), Medes (Me) and Scandola (Sc)) during the seasons indicated in columns. TRFs were designated by their size (in bp) after binning. The filling colors represent the relative fluorescence of the TRFs according to the range presented in the color key.

**Figure 4:**
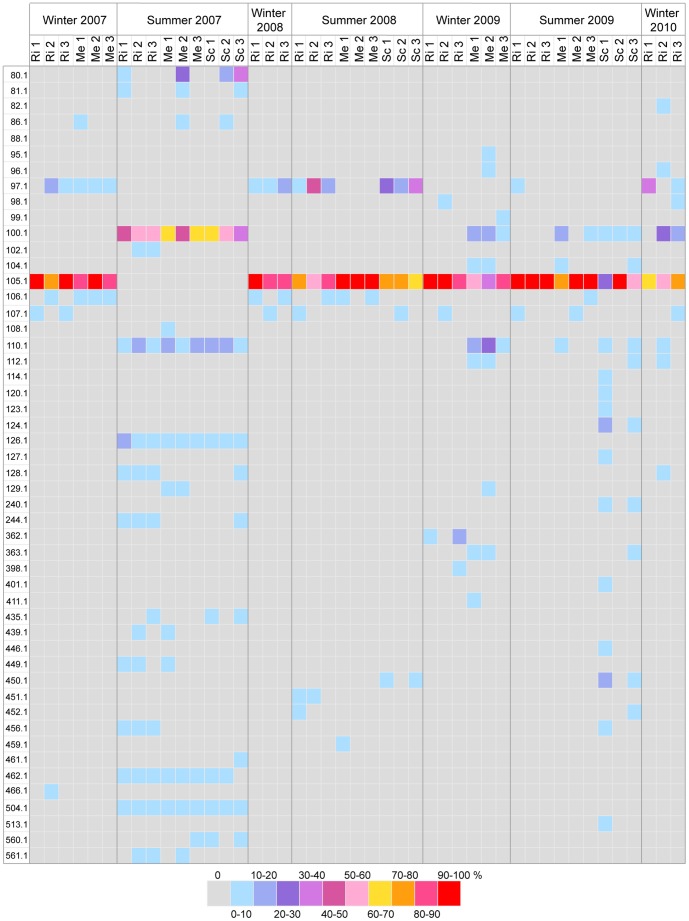
Relative abundances of *Msp*I TRFs in *P. clavata* samples analyzed by T-RFLP. As described in Figure 3, with the restriction enzyme *Msp*I.

Temporal and spatial changes in bacterial diversity associated with *P. clavata* were investigated by T-RFLP analysis. A total of 186 profiles, including run duplicates (96 and 90 profiles for *Cfo*I and *Msp*I digestion, respectively), were obtained from tissues of 48 *P. clavata* colonies that were seasonally sampled between March 2007 and September 2010 at the Riou, Medes and Scandola sites (3 colonies per site for each sampling). The profiles generated by run duplicates were consistent. The diversity and abundance of the detected TRFs are shown in Figures 3 and 4 for each restriction enzyme. In total, 57 different TRFs were observed upon *Cfo*I digestion (Figure 3), and 50 TRFs were observed for *Msp*I digests (Figure 4). None of the TRFs were shared by all samples, independent of the endonuclease. The T-RFLP profiles obtained for replicate colonies of each sampling were consistent, although variations in peak intensity were observed, likely due to the non-quantitative PCR amplification step. However, the dominant TRFs between replicates from the same seasonal sampling were broadly conserved between the *Cfo*I and *Msp*I data sets.

Two dominant TRFs were consistently recovered throughout most of the survey, indicating the presence of conserved ribotypes. The *Cfo*I profiles were dominated by TRF-171.4, with the exception of 9 samples from summer 2007 (Figure 3). When present, this TRF accounted for 37.3 to 91.1% of the total area of the TRF peaks in the fluorescence profiles. Profiles from *P. clavata* colonies sampled in summer 2007 did not contain TRF-171.4 but were dominated by TRF-193.4 (up to 87.0% of the profile's total fluorescence). These 2 TRFs were simultaneously detected in 15 of the *Cfo*I profiles of the survey, although TRF-171.4 was dominant in each case. The same trend was observed for the *Msp*I profiles (Figure 4). All but 9 profiles were dominated by TRF-105.1 (25.7 to 100% of the profile's total fluorescence). The 9 other profiles corresponded to colonies from summer 2007 and were dominated by another restriction fragment (TRF-100.1), with a relative fluorescence of 36.8 to 68.9%. In addition to TRF-100.1, 3 other notable TRFs common to the 9 *Msp*I profiles of summer 2007 were observed: TRF-110.1, TRF-126.1 and TRF-504.1. The last 2 TRFs seemed to be specifically associated with the summer 2007 samples, as they were not observed in any other profile. By contrast, the *Msp*I TRF-100.1 was detected in 10 other profiles from the 2009 and 2010 samples, among which 7 also contained TRF-110.1.

Overall, peak diversity was significantly higher in the summer 2007 profiles, with an average of 8.9 ± 2.5 *Cfo*I TRFs versus 5.9 ± 1.6 for other profiles and 9.3 ± 2.2 *MspI* TRFs versus 3.8 ± 2.8 for other profiles (p<0.001). The T-RFLP profiles of the bacterial communities in the surrounding seawater were compared with the profiles of colonies sampled on 3 occasions in Riou (winter 2007 and 2008 and summer 2007). None of the detected TRF peaks were common to the seawater and gorgonian tissue profiles (data not shown).

**Figure 5:**
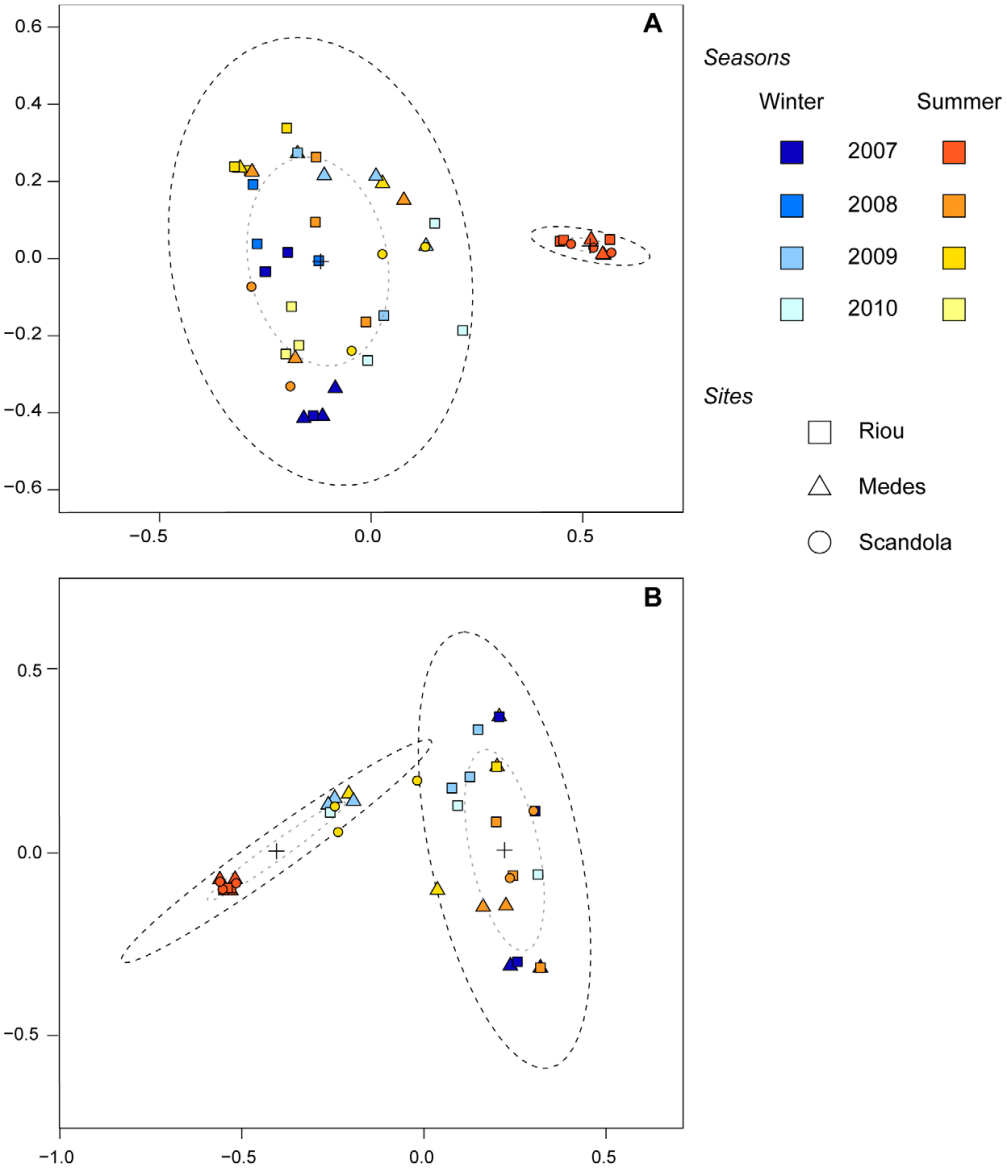
MDS ordination plot of *P. clavata* bacterial communities. 2-D scatter plots of T-RFLP profiles from *P. clavata* colonies sampled at 3 sites in winter and summer from 2007 to 2010 are based on the Dice similarity matrix for the T-RFLP data retrieved from the amplified bacterial 16S rDNA digested with *Cfo*I (A) and *Msp*I (B). Each symbol represents the bacterial community of an individual sample from Riou (square), Medes (triangle) or Scandola (circle), and the colors correspond to different sampling seasons. Sample clusters are based on coordinates determined with the k-means method. Dotted ellipses contain 50% (grey dots) or 95% (dark dots) of the points that contribute to the cluster. The centers of the confidence ellipses are identified with a cross.

The ordination of *P. clavata*-associated community profiles by MDS did not demonstrate visible clustering by sampling site (Figure 5) but revealed that most of the *Cfo*I (Figure 5A) and *Msp*I (Figure 5B) profiles grouped regardless of geographic origin. We observed that 32 (67%) of the *Msp*I profiles and 39 (81%) of the *Cfo*I profiles of the 48 *P. clavata* colonies sampled between March 2007 and September 2010 grouped together, thus suggesting the presence of similar bacterial assemblages at most sampling times. However, a tight and distinct clustering of the 9 *Cfo*I bacterial profiles from summer 2007 for the 3 sites was observed (Figure 5A). For the *Msp*I analysis (Figure 5B), the 9 samples from summer 2007 grouped with 7 other samples from Medes in summer and winter 2009, Riou in winter 2010 and Scandola in summer 2009. This difference in ordination of the *Cfo*I and *Msp*I bacterial profiles may have resulted from the common presence of 2 *Msp*I TRFs (TRF-100.1 and TRF-110.1) in these 7 latter samples and all summer 2007 samples, while a single *Cfo*I TRF (TRF-193.4) was shared between all summer 2007 samples.

### Phylogenetic analysis of 16S rDNA clone libraries

To determine if the variations in the dominant peaks observed by T-RFLP were associated with changes in the phylogenetic composition of the bacterial communities, bacterial 16S rDNA clone libraries were constructed from the *P. clavata* colonies sampled in Riou during winter 2007 and 2008 and in Riou, Medes and Scandola during summer 2007.

**Figure 6:**
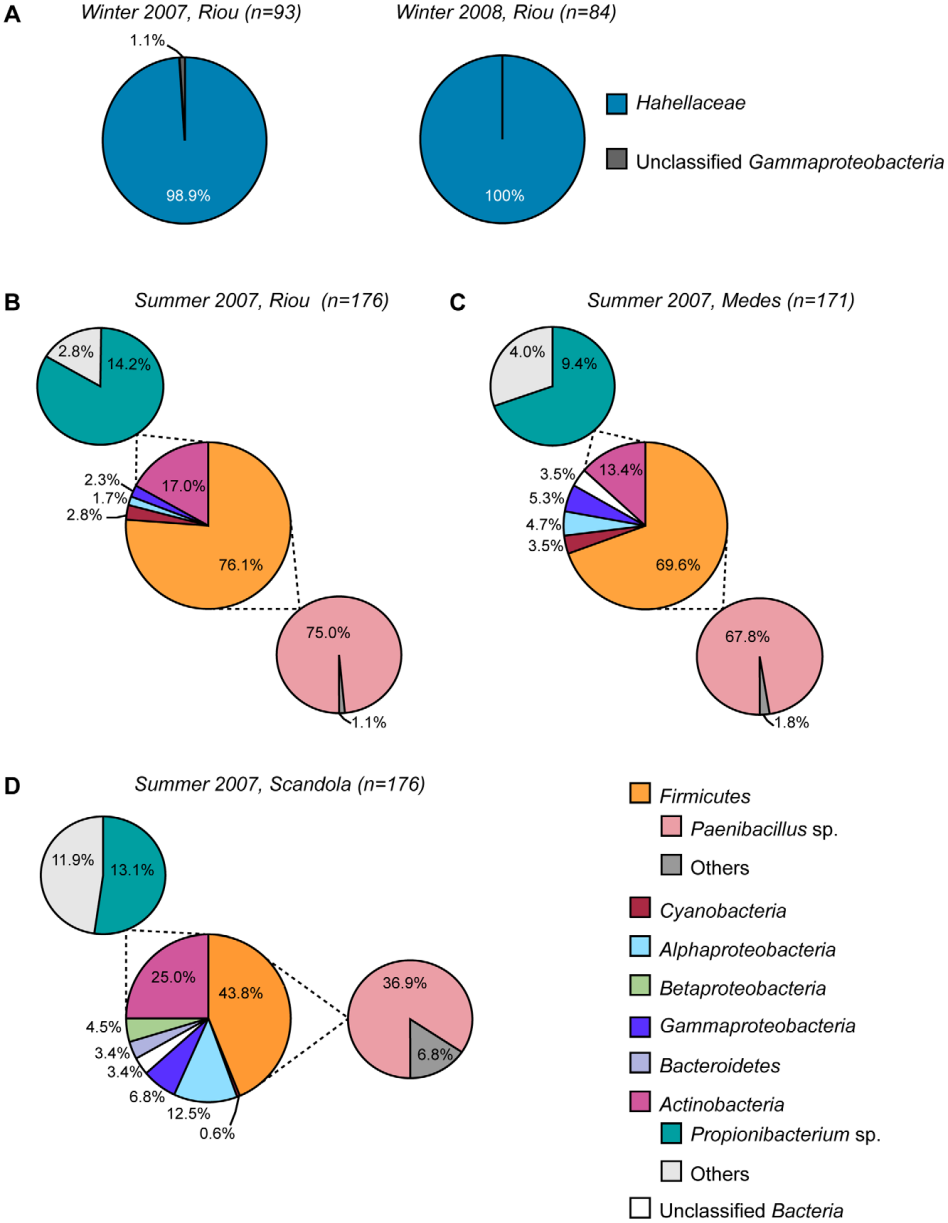
Bacterial diversity of clone libraries derived from *P. clavata* colonies. Pie charts illustrating the composition of bacterial communities in the winter 2007 and winter 2008 samples from Riou (A) and the summer 2007 samples from Riou (B), Medes (C) and Scandola (D). The large pies represent the clone library composition at the family level (A) and at the phyla or class levels (B–D). The smaller pies in B–D represent the affiliations of *Actinobacteria* and *Firmicutes* sequences at the genus level. The relative abundances of the bacterial groups retrieved from the libraries are indicated as percentages.

The library constructed from colonies sampled in winter 2007 in Riou was largely dominated by a single ribotype that accounted for 98.9% of the sequenced clones (Figure 6A). The winter 2008 library was exclusively composed of the same ribotype. This uncommon dominance is unlikely to be the consequence of a contamination bias because bacterial DNA extractions were performed separately, in March 2007 and February 2008, for the construction of the corresponding 16S rDNA libraries. The sequence divergence was very low (<1%) between clones belonging to this dominant ribotype in the 2 winter libraries, suggesting that it may represent microheterogeneity of 16S rRNA gene copies within a unique bacterial species. According to pairwise comparisons, this ribotype is related to members of the *Hahellaceae* family within the *Oceanospirillales* order (class *Gammaproteobacteria*). The closest cultivated strains were *Endozoicomonas elysicola* (AB196667), *Endozoicomonas montiporae* CL-33 (FJ347758) and *Spongiobacter nickelotolerans* (AB205011), with 92–93% 16S rDNA sequence similarity (see Figure S1 for a 16S rDNA-based phylogenetic tree). Although the *Hahellaceae*-related ribotype is undoubtedly a highly abundant component in the *P. clavata*-associated assemblage, a better description of diversity within these libraries would certainly require the analysis of a larger number of clones.

By contrast, the 16S rDNA sequences obtained from the summer 2007 clone libraries exhibited greater diversity and were distributed among 5 bacterial phyla, namely *Firmicutes*, *Actinobacteria*, *Proteobacteria*, *Bacteroidetes* and *Cyanobacteria* (Figure 6B–D). *Firmicutes* was dominant in each of the libraries, constituting 76.1% of the sequences from Riou, 69.6% from Medes and 43.8% from Scandola. The second most abundant phylum was *Actinobacteria*, which comprised 13.4% of the cloned sequences in the Medes library and 25.0% in the Scandola library. The *Gammaproteobacteria* were represented to a lesser degree in the bacterial community in each of the 3 summer 2007 libraries (2.3% to 6.8%), and none of these ribotypes were related to the *Hahellaceae* family. Analysis at lower taxonomic levels revealed the presence of a high proportion of almost identical sequences affiliated with the genus *Paenibacillus,* accounting for 84.4% to 98.6% of the *Firmicutes* species (36.9% to 75.0% of the clone libraries). According to BLAST and SeqMatch searches, the closest relative is *Paenibacillus validus* (AB073203), which shares >98% sequence similarity with this ribotype. A second dominant ribotype was identified within *Actinobacteria*, which represented 52.4% to 83.5% of this phylum in each of the libraries (9.4% to 14.2% of the total number of clones). The nearest relative of this group of sequences is *Propionibacterium acnes* (AE017283; >98% similarity for 16S rDNA). While *Paenibacillus-* and *Propionibacterium*-related sequences were predominant in the 3 libraries from summer 2007, the communities exhibited differing diversities. The Chao1 and Shannon-Weaver indices were highest in the Scandola library, although there is considerable overlap of the estimated total number of ribotypes at the 95% confidence interval within these 3 libraries (Table 1).

**Table 1:** Observed and estimated richness of the 16S rDNA clone libraries from the *P. clavata* samples in summer 2007.

Summer 2007 clone library	No. of clones analyzed	No. of ribotypes observed	Chao1 richness index	Shannon-Weaver diversity index
Riou	176	14	32 (18–95)	0.98
Medes	171	19	28 (21–59)	1.41
Scandola	176	38	49 (41–73)	2.67

Values were determined using a 97% sequence similarity threshold for ribotype groups. Chao1 values given in parentheses are 95% confidence intervals.

To identify the TRFs of the dominant ribotypes, the T-RFLP profiles of representative clones that were recovered from the 5 libraries were analyzed. The observed TRFs of the winter libraries clones carrying the *Hahellaceae* 16S rDNA sequences matched *Cfo*I TRF-171.4 and *Msp*I TRF-105.1, which correspond to the dominant peaks in all the *P. clavata* community profiles, with the exception of the summer 2007 samples (Figures 3 and 4). Clones corresponding to the *Paenibacillus* sequences in the summer 2007 libraries generated *Cfo*I and *Msp*I TRFs (TRF-193.4 and TRF-100.1, respectively) that matched the dominant peaks in the corresponding T-RFLP summer profiles. *Propionibacterium* 16S rDNA cloned sequences only generated an *Msp*I peak (TRF-126.1), and no signal was detected following *Cfo*I hydrolysis. *In silico* digestions of these *Propionibacterium*-related sequences showed that the size (636 bp) of the predicted *Cfo*I TRF was beyond our detection range.

## Discussion

This study provides the first description of the spatial and temporal patterns of the structure of the microbial communities associated with a temperate gorgonian in the Mediterranean Sea. The analysis of the DGGE fingerprint data indicated that the bacterial diversity hosted by *P. clavata* is broadly similar within and between individual colonies from a population and differs from the community composition within the surrounding water. In addition, the MDS ordination of the T-RFLP diversity profiles revealed a lack of clustering by location for the gorgonian populations sampled among our 3 study sites, which are separated by hundreds of kilometers. These results clearly suggest that the structure of bacterial communities that inhabit *P. clavata* does not rely on local environmental drivers or discrete gorgonian populations. Most of the T-RFLP profiles from the 48 colonies sampled between March 2007 and September 2010 grouped together, thus supporting the notion of specific bacterial-host associations. Taken together, data from the T-RFLP analysis and 16S rRNA gene clone libraries indicate that *P. clavata* colonies are associated with an almost permanent microbial population that is highly dominated by a unique gammaproteobacterial ribotype. However, we detected a transient compositional shift between this relatively stable bacterial assemblage that was observed throughout 7 of the 8 investigated seasons, with a strikingly different diversity pattern in summer 2007.

The dominant identified ribotype is affiliated with the family *Hahellaceae* within the order *Oceanospirillales* and is related to *Endozoicomonas* and *Spongiobacter* spp. Interestingly, a meta-analysis of coral-associated bacterial assemblages revealed that *Oceanospirillales-*affiliated sequences were among the most frequent ribotypes found in healthy hexacorals [50], and several recent reports indicated that *Hahellaceae* members may represent a common group of coral-associated *Oceanospirillales*. *Spongiobacter* sequences have been retrieved from the hexacoral *Acropora millepora,* in which they represent the largest proportion of bacterial clone libraries [28], [51]. In 2 other hard coral species, *Acropora hyacinthus* and *Stylophora pistillata*, Kvennefors *et al.* [29] identified a cluster of ribotypes that are closely related to the genus *Endozoicomonas*. This so-named “Type A Associates” cluster includes several ribotypes that were previously observed in different tropical coral species or specifically associated with benthic marine invertebrates. *Spongiobacter*-related 16S ribosomal sequences were also detected in the cold-water coral *Madrepora oculata* and dominated the DGGE banding pattern of the associated bacterial community in this species [52]. Very recently, Morrow *et al.* [53] found that *Endozoicomonas* represented the most abundant bacterial genus in communities of the reef-building coral *Porites astreoides*, representing up to 99% of the 16S rRNA sequences recovered from colonies in one of the studied sites. The presence of *Hahellaceae* as a major component of the bacterial communities in both hexacorals and gorgonians (octocorals) suggests that these associates have differentiated to form a stable symbiotic complex specific to a cnidarian taxon. Further research focusing on the patterns of diversity of this bacterial group will certainly help to understand the evolutionary forces that may drive host-associated community composition in divergent phylogenetic lineages of cnidarians.

In contrast to the unique *Hahellaceae* ribotype revealed by our study in *P. clavata*, several distinct *Endozoicomonas-Spongiobacter* sequences coexist in hexacoral species. This difference suggests that distinct hexa- and octocoral hosts may exert different selective controls on their microbial partners, resulting in various diversity patterns of *Hahellaceae* associates. Consistent with this hypothesis of host-driven control on associated *Hahellaceae* bacteria, the ribotype identified in *P. clavata* did not closely match the *Spongiobacter-Endozoicomonas* sequences found in hexacorals (<93% similar) but was more closely related (>96% similar) to bacterial sequences retrieved from the tropical gorgonian *Gorgonia ventalina* (GenBank accession number GU118518; [13]), suggesting that the species associated with *P. clavata* may belong to a new *Hahellaceae* genus that is adapted to gorgonian hosts.

To date, very little is known about the potential contribution of *Hahellaceae* bacteria to the functioning of the holobiont. One study has shown that *Spongiobacter*-related organisms isolated from *A. millepora* tissues possess the ability to metabolize the organosulfur compound dimethylsulfoniopropionate (DMSP), suggesting a role in sulfur cycling in scleractinian corals [51]. However, because DMSP production in corals is believed to rely on the presence of endosymbiotic zooxanthellae [51], a similar hypothesis for *Hahellaceae*-affiliated bacteria in *P. clavata* seems unlikely because this gorgonian species is devoid of photosynthetic symbionts [20]. Despite multiple trials, we failed to isolate culturable *Hahellaceae* bacteria from *P. clavata* tissues; consequently, their metabolic potential could not be investigated. Another interesting property of the members of the *Oceanospirillales* clade is the production of extracellular hydrolytic enzymes that are involved in the degradation of various complex organic substrates, providing a potential nutritional integration between bacterial associates and their host, as suggested for the endosymbiotic *Oceanospirillales* found in the deep-sea worm *Osedax* [54]. In the case of coral-associated bacteria, one might speculate that heterotrophic *Hahellaceae* help supply the host with macromolecular nutrients that are not directly assimilated under a complex form. The previous identification of *Gammaproteobacteria* aggregates within the gastrodermis tissue layer in the digestive cavity of several reef-building corals suggests that these bacteria may play such a role in the coral diet [55]. However, the available data on the 16S rRNA gene sequences of these intracellular bacteria are restricted to the *Gammaproteobacteria* FISH probe regions, thus precluding phylogenetic affiliation at the genus or family levels and hindering comparisons with *Hahellaceae* sequences. Further investigations, including *in situ* localization of bacteria in *P. clavata,* are clearly required to provide information on their possible role in the functioning of the holobiont. Hopefully, the 16S rRNA gene sequence retrieved from the dominant *Hahellaceae* ribotype in our study will allow us to design oligonucleotide probes for their specific detection in gorgonian tissues.

While our T-RFLP and clone libraries analysis indicated the existence of a relatively stable *Hahellaceae*-dominated microbiota in *P. clavata* populations across a large geographic range and throughout the 4-year survey, we identified a simultaneous and deep compositional shift of communities in summer 2007 at a regional scale. During this summer, the *Hahellaceae* sequences were not detected by either technique and were transiently replaced by more diverse assemblages with prominent *Paenibacillus-* and *Propionibacterium*-related sequences, 2 bacterial genera that respectively belong to *Firmicutes* and *Actinobacteria*. Members of *Firmicutes* and *Actinobacteria* have previously been detected in cold-water and tropical scleractinian corals [4], [29], [56], [57], where they are generally found in low abundance in healthy and/or bleached colonies [50] although representatives of *Paenibacillus* and *Propionibacterium* spp. were among the most commonly recovered bacteria from a Brazilian coral species [58]. Because we did not observe a similar transition in any of the other summers that were analyzed, we suggest that an abnormal and transient disruption of the host-*Hahellaceae* association occurred in summer 2007. However, ordination of the T-RFLP *Msp*I data set revealed that the profiles of all summer 2007 samples grouped with profiles from several colonies sampled in other seasons (Figure 5B). Significantly, the *Paenibacillus* TRF (TRF-100.1) was observed together with the dominant *Hahellaceae* TRF (TRF-105.1) in the latter profiles (Figure 4). These observations indicate a possible overlap between the 2 communities and suggest that bacteria related to *Paenibacillus* could be normal but infrequently detected associates, except during periods of anomalous abundance. Therefore, the shift in dominant ribotypes from *Hahellaceae* to *Paenibacillus* may result from a transient imbalance of endogenous bacterial populations that naturally reside in the holobiont rather than opportunistic colonization by *Paenibacillus* from the surrounding water concomitant with a decrease in the *Hahellaceae* community. The subsequent shift back toward the initial *Hahellaceae*-dominated community further supports the hypothesis that the holobiont strongly regulates its microbial diversity and indicates that *Hahellaceae* most likely represent host-specific bacterial associates of *P. clavata*.

The causes of the transition in *P. clavata* bacterial diversity from *Hahellaceae*- to *Paenibacillus-*dominant ribotypes during summer 2007 are not clear. As mentioned above, the shift occurred at the 3 studied areas in an almost synchronous manner, suggesting the involvement of environmental and/or biological factors acting on a large geographical scale. Several previous studies have identified shifts in coral-associated bacterial communities during bleaching and disease outbreaks or under conditions of environmental stress, such as increased temperature and organic matter enrichment [55], [59], [60]. For instance, changes in microbial associates were observed in the stony coral *A. millepora* during a mass bleaching event, and occurred prior to visual signs of bleaching on the sampled colonies [28]. Major shifts in bacterial communities were also observed in diseased colonies of 2 coral species that were affected by White Syndrome disease [29]. Interestingly, the *Hahellaceae*-related bacteria found in healthy colonies were replaced by other bacterial groups in bleached or diseased individuals. Whether the shift in bacterial assemblages is the cause or the effect of the disease is unclear, but these observations indicate that changes in *Hahellaceae*-host associations are related to an altered physiological state of the holobiont during stress conditions.

Regarding the potential causes of stress within the gorgonian populations in summer 2007, the available temperature time series data recorded at the study areas during the survey period (T-MedNet network; http://t-mednet.org) did not reveal any temperature anomaly. In addition, surveys of *P. clavata* populations conducted during and after summer 2007 in the 3 study areas did not reveal any symptoms of disease (J. Garrabou *et al.*, unpublished data). Notably, we did not detect the presence of *Vibrio coralliilyticus* 16S rDNA in the *Paenibacillus*-dominated summer 2007 clone libraries, although this *Vibrio* has been implicated in recent disease outbreaks in *P. clavata* populations during climatic anomalies [22], [23]. Several other *Vibrio* species were also involved in *P. clavata* tissue necrosis in Mediterranean [17], [61], or recovered from healthy and diseased cold-water gorgonians [62]. Altogether, this suggested that unhealthy conditions of the host are allowing colonization by potentially pathogenic *Vibrio* strains that can also be a natural component of the holobiont and may exploit the disturbance of the normal microbiota [63]. By contrast, we detected only 2 *Vibrionaceae* sequences among the 523 clones that were analyzed from the summer 2007 libraries (data not shown). This result suggests a very moderate abundance of *Vibrio* and indicates that the shift observed in *P. clavata* was likely not related to the onset of a disease. However we cannot rule out that other bacterial groups among the taxon diversity in summer 2007 may represent potential pathogens or detrimental associates that overcome the *Hahellaceae*-dominated natural assemblage. For instance, although *Paenibacillus* and *Propionibacterium* spp. are not considered as coral pathogens and can be normal components of the associated microbiota, their overwhelming dominance in summer 2007 might have resulted in transiently compromised holobiont homeostasis without expression of disease symptoms.

Besides climate-related stresses and disease, regional oceanographic perturbations including land run-off and anthropogenic disturbance have also been shown to cause changes in microbial communities associated with corals [56], [64], [65]. Degraded ecological conditions may facilitate the existence of alternate states of microbial community structure and overgrowth of opportunistic bacteria [59]. However, an exposure of *P. clavata* colonies to a pollutant or effluent in summer 2007 is very unlikely considering the large geographical area in which this bacterial shift was observed. In addition, our studies sites are variably impacted by anthropogenic effects as the 3 regions are subjected to different levels of protection. Riou site was located in a non-protected region, a few kilometers away from Marseille, the second major French city. Medes and Scandola sites are both located in marine protected areas, Scandola being a no-take area submitted to very limited human pressure. Therefore, local anthropogenic impacts do not seem to be the cause for the bacterial shifts simultaneously observed in the 3 sites in summer 2007, although a monitoring of environmental parameters and microbial community structure in the water column would be required to better evaluate differences between the sites. Finally, the factors that could have directly or indirectly caused a transient change in the natural bacterial community remain unclear and several other hypotheses cannot be ruled out, such as a bacteriophage infection targeting *Hahellaceae* and causing microbial mortality [66], or the occurrence of subtle alterations in gorgonian physiology in summer [67] that would disrupt the host-bacteria relationship without macroscopic disease signs.

In conclusion, the present work provides a reliable evaluation of the structure of and variations in gorgonian-associated bacterial communities. To our knowledge, this is the first spatiotemporal study demonstrating that transient but dramatic shifts in the natural baseline of these assemblages can arise in apparently healthy octocorals. In light of our results, it is important to encourage multi-year monitoring to avoid erroneous or incomplete descriptions of the bacterial assemblages, as we might have drawn by investigating the *P. clavata* microbiota during the phase shift. Although this shift was only observed once in the 4-year survey and thus presumably corresponds to an abnormal or stressful event, it indicates that transient bacterial populations may replace the natural *Hahellaceae*-dominated community without visible evidence of deleterious effects. Further studies are now required to address the question of whether the phase shift in the microbial community may represent a potential monitoring tool for determining the health state of gorgonians and the ecological condition of their environment. This research may aid in understanding the causes of recurrent mass mortalities and designing effective management strategies to preserve one of the most emblematic Mediterranean species.

## Supporting Information

Figure S1 **Phylogenetic relationships between the dominant** ***P. clavata*** **-associated ribotype and its closest relatives.** Neighbor-joining tree based on 16S rRNA gene sequences filtered to ∼720 aligned nucleotide positions. Representative clones retrieved from *P. clavata* libraries in winter 2007 and winter 2008 are marked in red, and the GenBank accession numbers of reference strains are shown in parentheses. Branch points supported by boostrap values >50% or >95% (based on 1000 resamplings) are indicated by open and filled circles, respectively. The *Gammaproteobacteria Pseudomonas aeruginosa* ATCC 10145 was used as an outgroup. Scale bar represents 0.02 changes per nucleotide.
(TIF)
