# Supplementary figures and images for: Transient Shifts in Bacterial Communities Associated with the Temperate Gorgonian Paramuricea clavata in the Northwestern Mediterranean Sea
Source: PLoS One. 2013 Feb 20;8(2):e57385. doi: 10.1371/journal.pone.0057385 (PMC3577713; doi:10.1371/journal.pone.0057385)

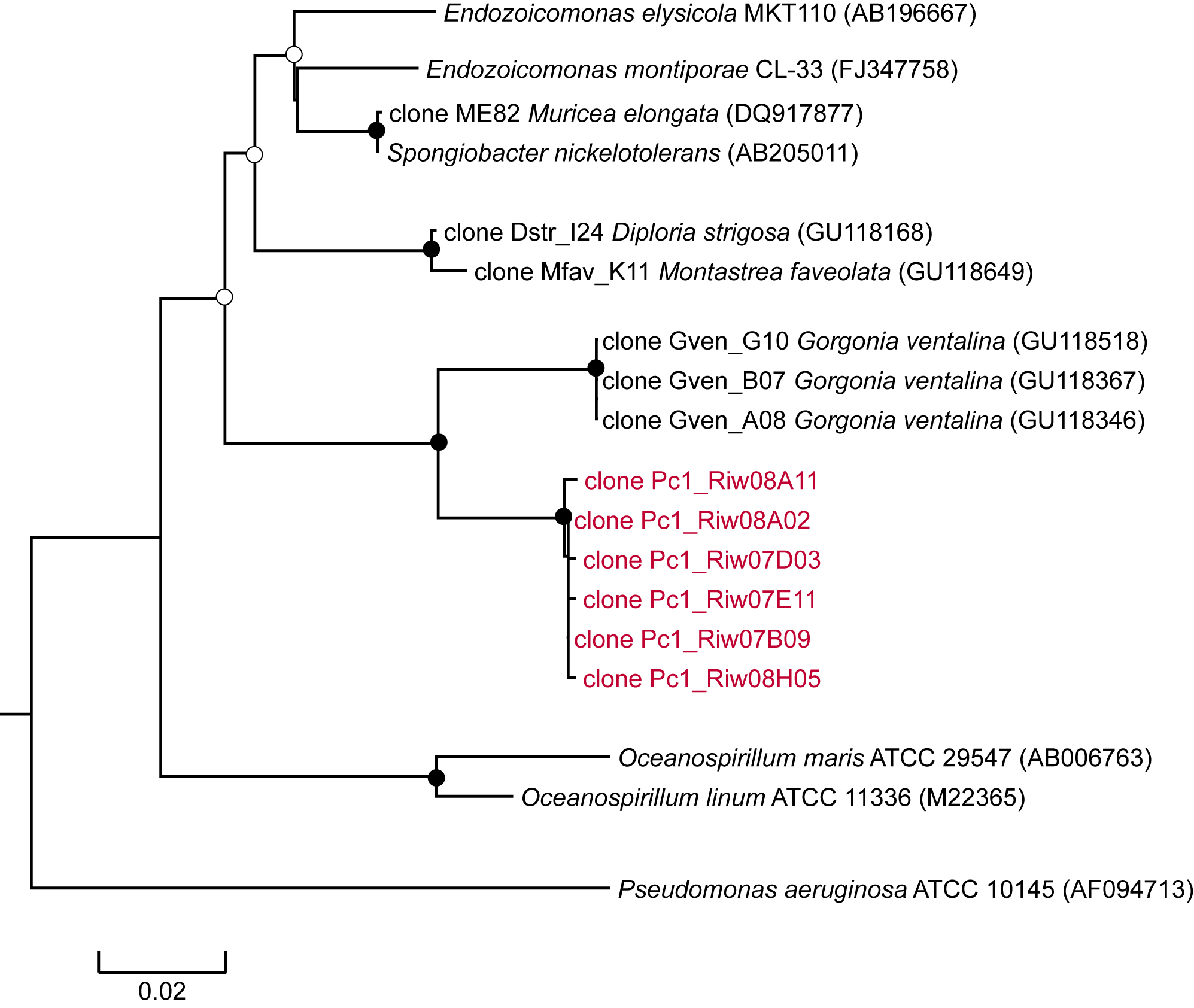

Supplement: Figure S1 — Phylogenetic relationships between the dominant P. clavata -associated ribotype and its closest relatives. Neighbor-joining tree based on 16S rRNA gene sequences filtered to ∼720 aligned nucleotide positions. Representative clones retrieved from P. clavata libraries in winter 2007 and winter 2008 are marked in red, and the GenBank accession numbers of reference strains are shown in parentheses. Branch points supported by boostrap values >50% or >95% (based on 1000 resamplings) are indicated by open and filled circles, respectively. The Gammaproteobacteria Pseudomonas aeruginosa ATCC 10145 was used as an outgroup. Scale bar represents 0.02 changes per nucleotide. (TIF) [file pone.0057385.s001.tif]
